# gga-miR-375 Plays a Key Role in Tumorigenesis Post Subgroup J Avian Leukosis Virus Infection

**DOI:** 10.1371/journal.pone.0090878

**Published:** 2014-04-02

**Authors:** Hongxin Li, Huiqing Shang, Dingming Shu, Huanmin Zhang, Jun Ji, Baoli Sun, Hongmei Li, Qingmei Xie

**Affiliations:** 1 College of Animal Science, South China Agricultural University, Guangzhou, P R China; 2 Key Laboratory of Chicken Genetics, Breeding and Reproduction, Ministry of Agriculture, Guangzhou, P R China; 3 State Key Laboratory of Livestock and Poultry Breeding, Guangzhou, P R China; 4 United States Department of Agriculture (USDA), Agriculture Research Service, Avian Disease and Oncology Laboratory, East Lansing, Michigan, United States of America; Fudan University, China

## Abstract

Avian leukosis is a neoplastic disease caused in part by subgroup J avian leukosis virus J (ALV-J). Micro ribonucleic acids (miRNAs) play pivotal oncogenic and tumour-suppressor roles in tumour development and progression. However, little is known about the potential role of miRNAs in avian leukosis tumours. We have found a novel tumour-suppressor miRNA, gga-miR-375, associated with avian leukosis tumorigenesis by miRNA microarray in a previous report. We have also previously studied the biological function of gga-miR-375; Overexpression of gga-miR-375 significantly inhibited DF-1 cell proliferation, and significantly reduced the expression of yes-associated protein 1 (YAP1) by repressing the activity of a luciferase reporter carrying the 3′-untranslated region of YAP1. This indicates that gga-miR-375 is frequently downregulated in avian leukosis by inhibiting cell proliferation through YAP1 oncogene targeting. Overexpression of gga-miR-375 markedly promoted serum starvation induced apoptosis, and there may be the reason why the tumour cycle is so long in the infected chickens. In vivo assays, gga-miR-375 was significantly downregulated in chicken livers 20 days after infection with ALV-J, and *YAP1* was significantly upregulated 20 days after ALV-J infection (*P*<0.05). We also found that expression of cyclin E, an important regulator of cell cycle progression, was significantly upregulated (*P*<0.05). *Drosophila* inhibitor of apoptosis protein 1 (DIAP1), which is related to caspase-dependent apoptosis, was also significantly upregulated after infection. Our data suggests that gga-miR-375 may function as a tumour suppressor thereby regulating cancer cell proliferation and it plays a key role in avian leukosis tumorigenesis.

## Introduction

Subgroup J avian leukosis virus (ALV-J), belonging to the family *Retroviridae*, subfamily *Orthoretrovirinae* and genus *Alpharetrovirus*, causes a variety of tumours in chickens. ALV-J was first isolated from meat-type chickens in Great Britain in 1988 [1]. In poultry, ALV-J spreads widely and induces myeloid leukosis (ML) and other tumours [2], [3], [4]. To date, infection of ALV-J in both commercial and meat-type chickens has caused major economic loss and seriously threatened the prosperity of the poultry industry all over the world [5], [6], [7]. It was reported, for instance, that ALV-J causes up to 60% morbidity and 20% mortality in some Chinese flocks [8]. Emerging ALV-J infections coarsely induce various tumours in both commercial laying hen flocks as well as native Chinese breeds of chickens [8], [9], which results in late onset and acute tumours in the field [10], [11].

Micro ribonucleic acids (miRNAs) are a class of small, non-coding RNAs consisting of 19–23 nucleotides, which are believed to play important roles in regulating various biological processes including tumorigenesis. It is presumed that miRNAs may eventually be instrumental in the diagnosis and treatment of cancer [12], [13], [14]. Since the discovery of the first miRNA, lin-4, in *Caenorhabditis elegans* two decades ago [15], there has been over 24,000 curated miRNA entries identified from various species (http://www.mirbase.org/). The expression of miRNAs has been profiled widely and aberrant miRNA expression has been reported to contribute to cancer development and progression [16], [17], [18], [19]. It was estimated that miRNAs are involved in the regulation of more than 30% of all protein-coding genes. It was also suggested that more than 50% of miRNA genes reside in cancer-associated genomic regions or in fragile sites [20], [21]. miRNAs offer a fast energy-saving and fine-tuning mechanism for translation control of protein production [22].

miR-375 was originally reported in pancreatic islets of humans and mice to regulate insulin secretion in isolated pancreatic cells [23]. Later, it was reported that miR-375 is commonly downregulated in human tumour tissues, which significantly increased cancer cell development [24], [25]. miR-375 was proposed as a candidate tumour suppressor miRNA in gastric carcinoma targeting 14-3-3zeta, Janus kinase 2, and phosphoinositide-dependent kinase 1 [24], [25] and was recognized to inhibit neuritis differentiation by lowering HuD levels [26]. In hepatocellular carcinoma (HCC) research, miR-375 was found as an important regulator of the yes-associated protein (YAP) oncogene with a potential therapeutic role in HCC treatment [27]. miR-375 promotes palmitate-induced lipoapoptosis in insulin-secreting NIT-1 cells through the inhibition of myotrophin (V1) protein expression [28]. Studies show that microRNAs are also involved in various diseases in poultry including avian influenza, avian leukosis, infectious bursal disease, Marek's disease, and ovarian carcinoma [29], [30], [31], [32], [33], [34].

ALV-J infected broilers are pathologically characterized with clearly visible grey-white nodules on the liver, spleen, and kidney. The nodules range widely in size and can be several times the size of the liver or spleen [35]. Although some signalling molecules have been uncovered that control stem cell proliferation, little is known about the molecular mechanism underlying ALV-J induced tumorigenesis and few prognostic markers have been identified that can predict genetic resistance or susceptibility to ALV-J in poultry. Our previous study has shown that gga-miR-375 to be frequently downregulated in the livers of chickens 10-weeks post ALV-J infection [35]. We also found that gga-miR-375 targets YAP1. Our findings, to some extent, were in agreement with a report on miRNA-375 in which it was shown to target the Hippo signalling effector YAP in human liver cancer and to inhibit tumour propagation [27]. The Hippo pathway was initially identified in flies and was implicated in controlling organ size. Hippo pathway's downstream target genes, such as *cyclin E* and *DIAP1*, are closely related to tumour suppression activities [36], [37], [38], [39].

The antiapoptotic properties and abnormal cell cycle progression are striking features of tumour cells. Overwhelming evidence indicates that aberrant miRNA expression is a cause or indicator of many disease processes. This study was undertaken to explore the roles of gga-miR-375 in chickens with respect to tumour development and progression induced by ALV-J infection. We also intended to elucidate the molecular mechanisms underlying tumorigenesis and to evaluate whether gga-miR-375 expression levels could serve as a biomarker for diagnostic purposes.

## Materials and Methods

### Virus and cell lines

The NX0101 strain of ALV-J used in all the relevant experiments and was obtained from Professor Cui, Shandong Agricultural University, People's Republic of China. DF-1 was an immortalized chicken embryo fibroblast cell line, and CHO was a continuous cell line of Chinese hamster ovary. DF-1 cell line was cultured in Dulbecco's modified eagle medium (DMEM) supplemented with 10% fetal bovine serum (FBS; Invitrogen Gibco Co, Carlsbad, CA, USA). CHO cell line were cultured in Roswell Park Memorial Institute (RMPI) 1640 supplemented with 10% FBS (Invitrogen Gibco Co).

### RNA oligoribonucleotides and cell transfections

The RNA duplex mimic chicken encoded miRNAs (see Table 1 for sequences) were designed as described previously [40]. The control RNA duplex (named gga-miR-NC; sense strand: UUCUCCGAACGUGUCACGUTT) was nonhomologous to any chicken genome sequence and used for gga-miR-375. All RNA oligoribonucleotides were purchased from Genepharma (Invitrogen Gibco Co). Transfection of RNA oligoribonucleotide(s) was done using X-tremeGENE siRNA Transfection Reagent (Roche Applied Science, Mannheim, Germany) following the manufacturer's protocol. For each transfection, 40 nM of RNA duplex were respectively used in a 6-well plate, unless otherwise indicated.

**Table 1:** Sequences of RNA oligonucleotides.

Name	Sense Strand/Sense Primer (5'-3')	Antisense Strand/Antisense Primer (5'-3')
Primers for Gene or 3'UTR Cloning		
YAP1	TTCTCGAGGGAGATGGGATGAATATAGAAGG	GGTGTCTAGACCACAGGCAGCAGGAGAC
YAP1-3'UTR	TTATCCCTCCTTTAAGTGAGATTCTCACAATTG	TTAAAGGAGGGATAAAGGAGTTATGGGT
Primers for qRT-PCR		
*YAP1* primers	GAACTCAGCATCAGCCATGA	CTACGGAGAGCCAATTCCTG
*Cyclin E* primers	CACCCTCTCCTGCAACCTAA	TGGTGCAACTTTGGTGGATA
*DIAP1* primers	GCCATAACAACTGCTGCTGA	TCTCTTTCAAGGCAGGCAAT
*GAPDH* primer	AGGCTGAGAACGGGAAACTTG	CACCTGCATCTGCCCATTTG

### Cell proliferation assay

Cell proliferation was measured using the WST-1 (Water-soluble tetrazolium, the sodium salt of 4-[3-(4iodophenyl)-2-(4-nitrophenyl)-2H-5-tetrazolio]-1, 3-benzene disulfonate; Roche Applied Science) colorimetric assay. Approximately 24 hours after transfection with gga-miR-375 or negative control oligonucleotides gga-miR-NC (miR-NC), DF-1 cells (1.0 × 10^5^ per millilitre) were seeded, respectively, into a 96-well plate and incubated for another 24, 48, or 72 hours. In addition, a non-transfected (mock) group was used as an additional control. Then, 10 μL of WST-1 reagent was added and incubated for 2 hours at 37°C. Absorbance was subsequently determined at wavelengths of 450 nm using multi-mode microplate readers (BioTek, Gene Company limited, Hong Kong, People's Republic of China). At least eight replicate wells were included for each experimental group, and all experiments were repeated independently three times. Cell proliferation was calculated by subtracting the absorbance values of the samples from the media alone (background level). The relative cell proliferation was normalized to the respective control.

### Colony formation assay

Approximately 24 hours after transfection with gga-miR-375 or mir-NC, 1,000 transfected DF-1 cells were seeded in 6-well plates and maintained in DMEM containing 10% FBS for 2 weeks. Moreover, a mock group was set as another control. Colonies were fixed with methanol and stained with 0.1% crystal violet in 20% methanol for 15 minutes.

### Wound healing assay

For the wound healing migration assay, approximately 24 hours after transfection with gga-miR-375 or miR-NC, DF-1 cells (1.6 × 10^5^ per millilitre) were seeded on 24-well plates. A mock group was also set. Forty-eight hours after transfection, a scratch wound was made on a confluent monolayer culture of DF-1 cells with a 100 mL pipette tip and fresh media was added for incubation for another 48 hours. The cells were imaged at three different time points (0, 24, and 48 hours after wound induction) using an inverted microscopy system (Leica DM IL LED, Leica Microsystems GmbH, Wetzlar, Germany) equipped with ProgResH MF camera (Jenoptik GmbH, Jena, Germany). The percentage of wound closure (cell migration) was calculated as relative wound area at a given time point normalized to wound area at 0 hours. All experiments were performed independently in triplicate.

### Apoptosis assays

Apoptosis was evaluated by apoptotic morphology and Annexin V-fluorescein isothiocyanate/propidium iodide (FITC/PI) assay for which cells were treated in similar ways as for the cell proliferation assay. About 24 hours after transfection with gga-miR-375 or miR-NC, DF-1 cells (1.0 × 10^5^ per millilitre) were seeded respectively into a 6-well plate and incubated for another 24, 48, or 72 hours under serum starvation; a blank control was also used. Then, Annexin V-FITC/PI assay (BD Biosciences Pharmingen, Franklin Lakes, NJ, USA) was performed according to the manufacturer's protocol. After staining, cells were analysed by FACS Calibur (Becton Dickinson, San Jose, CA, USA). For morphologic examination, after 48 hour serum starvation treatment, cells were stained with 4′-6′-diamidino-2-phenylindole (DAPI; Sigma-Aldrich Co, St Louis, MO, USA) and those with fragmented or condensed nuclei in deep staining were counted as apoptotic cells. At least 500 cells were counted for each plate. The background luminescence associated with cell culture and assay reagent (blank reaction) was subtracted from the experimental value.

### Vector construction

To construct a luciferase reporter vector, pmiRGLO-YAP1-3′UTR-wt, a wild-type 3′ UTR fragment of YAP1, was amplified by RT-PCR using the primers 5′-TTCTCGAGGGAGATGGGATGAATATAGAAGG-3′ and 5′-GGTGTCTAGACCACAGGCAGCAGGAGAC-3′. The putative binding sites for gga-miR-375 was inserted downstream of the stop codon of firefly luciferase in pmiRGLO Dual-Luciferase miRNA Target Expression Vector (Ambion, Promega, Beijing, People's Republic of China) as described previously[41] (designated as YAP1'UTR-wt). PmiRGLO-YAP1-3′UTR-mut, which carries a mutated sequence in the complementary site for the seed region of gga-miR-375, was generated using the primers 5′-TTATCCCTCCTTTAAGTGAGATTCTCACAATTG-3' and 5'-TTAAAGGAGGGATAAAGGAGTTATGGGT-3' (designated as YAP1-3′UTR-mut).

### Dual luciferase reporter assay

Dual luciferase reporter assay was comprised of two reporters; one is Renilla luciferase expression construct, the other is a firefly luciferase expression construct in pmiRGLO containing the assayed 3′UTR sequences. For luciferase reporter assay, CHO cells (3.4 × 10^5^) were plated in a 24-well plate and then co-transfected with 10 nmol/L gga-miR-375 or miR-NC, 20 ng YAP1-3′UTR-wt, or YAP1-3′UTR-mut, and 4 ng pRL-TK (Promega) using X-tremeGENE siRNA Transfection Reagent (Invitrogen Roche Applied Science) following the manufacturer's protocol. Cells were collected 48 hours after transfection and analysed using the Dual-Luciferase Reporter Assay System (Promega). Luciferase activity was detected by Lumat LB 9507 Ultra Sensitive Tube Luminometer (Titertek Berthold, Nanjing, People's Republic of China). Firefly luciferase activity of each sample was normalized by Renilla luciferase activity. Transfections were done in duplicates and repeated independently at least three times.

### Western blotting

At 48 or 72 hours after transfection with gga-miR-375 or miR-NC, DF-1 or CHO cells were subjected to Western blot analysis as described previously [42]. In addition, a non-transfected (mock) group was set. The primary antibodies used for Western blot analysis were polyclonal rabbit anti anti-YAP1 (1∶600; predicted molecular weight: 65 kDa; Bioss Inc, Wobourn, MA, USA) and β-actin (1∶600; predicted molecular weight: 42 kDa; Bioss Inc) which served as a protein loading control. Secondary antibody was goat polyclonal anti rabbit IgG (H+L)-horseradish peroxidase (HRP; Bioss Inc).

### Animal experiment

Specific pathogen-free (SPF) chickens were purchased from Guangdong Wen's Foodstuffs Group Co Ltd (Yunfu, People's Republic of China), housed in negatively-pressured biosecurity isolators under quarantine conditions, and provided with water and commercial feed ad libitum. One hundred and one day old SPF chickens were randomly divided into two groups of fifty chickens each. The first group (NX0101) was inoculated intra-abdominally at 1 day of age with 10^3.7^ TCID_50_/0.2 mL virulent NX0101 strain. The other group (NC) was inoculated with the same volume of nutrient solution. The second group was used as the control group. Three chickens from each of the two groups were euthanized for necropsy every 10 days post infection. Tissues samples were collected from each chicken at necropsy and snap frozen in liquid nitrogen. The tissue samples were stored at −70 °C until subsequent analysis. Institutional and national guidelines for the use and care of experimental animals were closely followed. Use of animals in this study was approved by the South China Agricultural University Committee of Animal Experiments (approval ID 201004152).

### Extraction of total RNA and miRNA

Total RNA was extracted from tissue samples with TRIzol reagent (Invitrogen); miRNA was extracted using the mirVana miRNA Isolation Kit (Life Technologies, Calrsbad, CA, USA) following the manufacturer's instructions.

### miRNA microarray

Microarray analysis was performed as described previously [24]. High-quality total RNAs, isolated from three chickens for each of the two groups (infection group and control group) at 10 weeks of age and three normal liver tissues using TRIzol reagent according to the manufacturer's instructions (Invitrogen), was carried out using the μParaflo microfluidic technology according to the manufacturer's protocol (LC Sciences, Houston, TX, USA).

### Real-time quantitative RT-PCR

gga-miR-375 and related gene expression was evaluated for absolute quantification using real-time quantitative reverse transcriptase polymerase chain reaction (RT-PCR) assays. gga-miR-375 and reference 5S rRNA, or the target genes and the reference gene glyceraldehyde-3-phosphate dehydrogenase (GAPDH) were amplified, cloned, and used as standard controls to generate standard curves following a previously described protocol [42]. The data from the real-time quantitative RT-PCR were analysed as relative miRNA expression using the 2^–△△^Ct method. The 5S rRNA was used as an internal control.

### Statistical analysis

Fixed effect was assessed by one-way analysis of variance (ANOVA). Unless otherwise noted, pairwise comparisons were done using Student's two-tailed t-test, and the differences were assessed by one-way analysis of variance (ANOVA) when more than two groups were compared. Results are presented as mean ± standard error of the mean (SEM) unless otherwise noted. The differences between groups were analysed when two or more groups were compared. Differences were considered statistically significant when *P*<0.05.

### Accession number

The microarray data were MIAME compliant and our data have been deposited in a MIAME compliant database (ArrayExpress, GEO ID: GSE28434). The sequences of gga-miR-375, hsa-miR-375 and rno-miR-3375 (MI0003705, MI0000783, MI0006140) described in this paper have been deposited in miRBase (http://www.mirbase.org/).

## Results

### Expression of gga-miR-375 in the liver of ALV-J infected chickens

**Figure 1:**
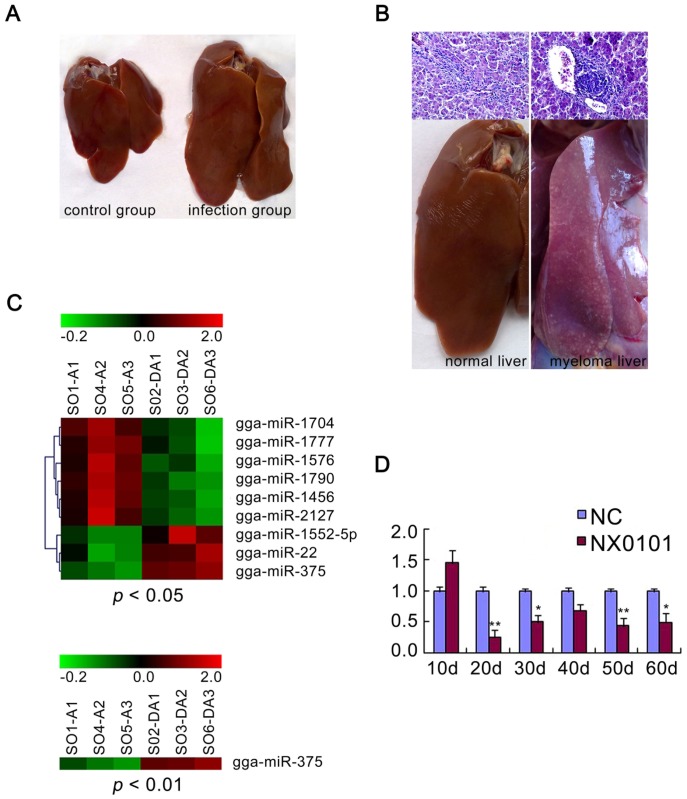
gga-miR-375 expression was frequently downregulated in ALV-J induced cancer. Liver lesions induced by viral infection in SPF white leghorn chickens at 70 days (**A**) and later (**B**). Representative histological features of nontumour liver and myeloma liver are shown with hematoxylin and eosin staining, 400× (**B**). (**C**) The miRNAs significantly associated with ALV-J by significance analysis of microarrays are listed. gga-miR-375 is most significantly associated with ALV-J infected liver tissue, as determined by significance analysis of microarrays. (**D**) Quantitative real-time PCR quantification of gga-miR-375 expression in the liver of ALV-J infected chickens every 10 days between 10 and 60 days post transfection (**P < 0.01, *p<0.05).

Compared to control chickens, most chickens in the ALV-J infected group showed gradual emaciation. Livers of the infected chickens were evidently bigger than the control group at 10 weeks (Figure 1A), and some developed tumour formations (Figure 1B). miRNA microarray profiling was performed in SPF chicken livers of controls and animals infected with ALV-J NX0101 strain, and the results showed that gga-miR-375 was significantly downregulated in SPF chicken livers of infected chickens at 10 weeks (*P*<0.01; Figure 1 C). In Animal experiments, the gga-miR-375 was significantly downregulated in liver tissue from the ALV-J infected chickens from 20 days post infection (Figure 1D), which may serve as a biomarker for diagnostic purposes.

### Overexpression of gga-miR-375 inhibited DF-1 cell proliferation and invasion

**Figure 2:**
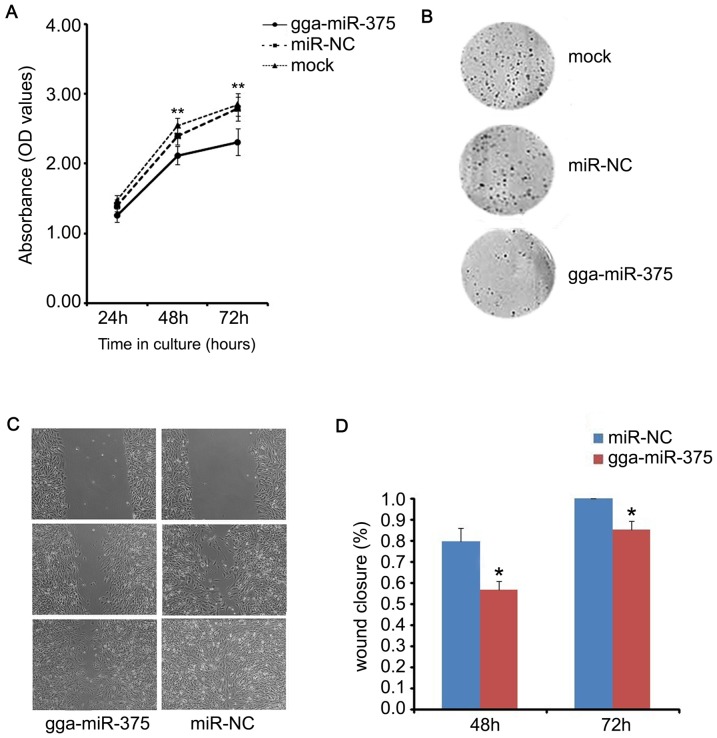
gga-miR-375 inhibited DF-1 cell proliferation and invasion. The cells transfected with gga-miR-375, miR-NC, or mock were subjected toWST-1 analysis, colony formation, and wound healing assay. (**A**) Effects of gga-miR-375 on proliferation over different time periods. Plotted means and standard errors were computed from data of three independent experiments; bars, SEM. **P<0.01. (**B**) Effects of gga-miR-375 on colony formation of DF-1 cells. (**C**) Images of cell migration from wound healing assay. Scratch wounds were made on confluent monolayer cultures 48 hours post transfection. Images of wound repair were taken at 0, 24, and 48 hours after wound. (D)The percentage of wound closure was normalized to the wound area at hour 0 (above panel). Plotted means and standard errors were computed from data of three independent experiments. The comparisons were evaluated using t-test; bars, SEM. *P<0.05.

To explore the role of gga-miR-375 in ALV-J carcinogenesis, we examined the effect of gga-miR-375 overexpression on the proliferation of DF-1 cell lines. The cells were transfected with either gga-miR-375 (gga-miR-375) or negative control oligonucleotides gga-miR-NC (miR-NC), and then cultured for various periods of time (24, 48, or 72 hours). In addition, a NT (mock) group was set as another control. Cell proliferation reagent WST-1 assays showed that all three groups (mock, miR-NC, and gga-miR-375) displayed fewer cells and overexpression of gga-miR-375 significantly inhibited the proliferation of DF-1 cells from 48 hours after transfection (Figure 2A) compared to the NC (miR-NC) or the mock group. Colony formation assay confirmed this inhibition (Figure 2B). To determine the effect of gga-miR-375 on the invasion of DF-1 cells, we conducted a wound healing assay. This assay showed that the invasion of the gga-miR-375 transfected cells was slower than the NC and non-transfected (NT) treated cells (Figure 2C, 2D). These results suggested that gga-miR-375 inhibits cell proliferation and invasion.

### gga-miR-375 promotes serum starvation induced apoptosis

**Figure 3:**
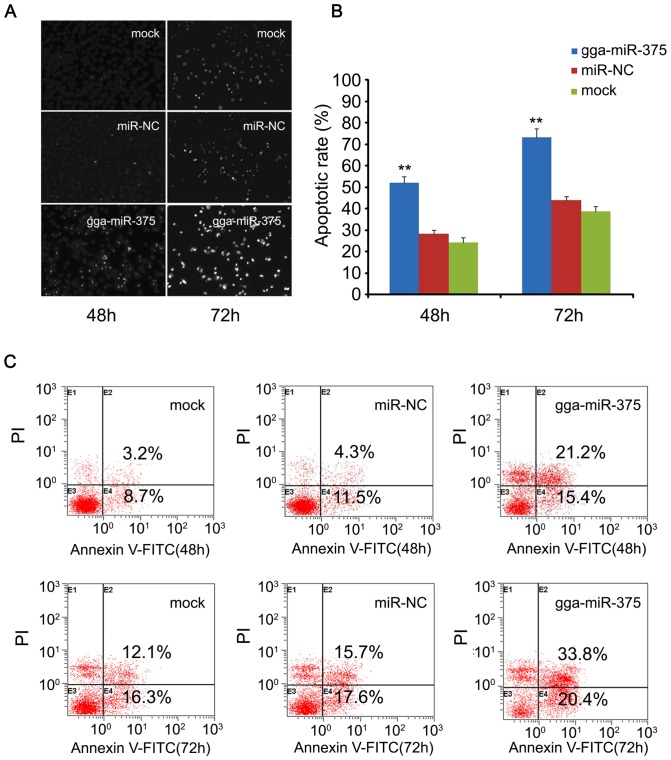
gga-miR-375 promoted serum starvation induced apoptosis. The cells transfected with gga-miR-375, miR-NC, or mock were subjected to DAPI and Annexin V-FITC/PI staining. (**A**) Apoptotic rates of DF-1 cells were evaluated by apoptotic morphology examination; (**C**) Apoptotic rates of DF-1 cells evaluated by Annexin V-FITC/PI staining during 48 and 72 hours post-transfection. (**B**) Apoptotic rate plot showing differences between gg-miR-375, NC, and mock treatment groups. Plotted means and standard errors were computed from data of three independent experiments; bars, SEM. **P<0.01.

Approximately 24, 48 and 72 hours after transfection, apoptosis was assessed by morphological examination and Annexin V-FITC/PI staining. The DAPI staining data suggests that gga-miR-375 overexpression remarkably increased serum starvation induced apoptosis in DF-1 cells (*P*<0.001; Figure 3A, 3B) at 48 and 72 hours. The analysis of Annexin V-FITC/PI staining confirmed the gga-miR-375 increased serum starvation induced apoptosis from 54.2% to 36.6% (Figure 3C). These results collectively demonstrate that gga-miR-375 may inhibit cell proliferation and invasion by increasing apoptosis under serum starvation.

### gga-miR-375 represses YAP1 protein production through 3′-UTR binding

**Figure 4:**
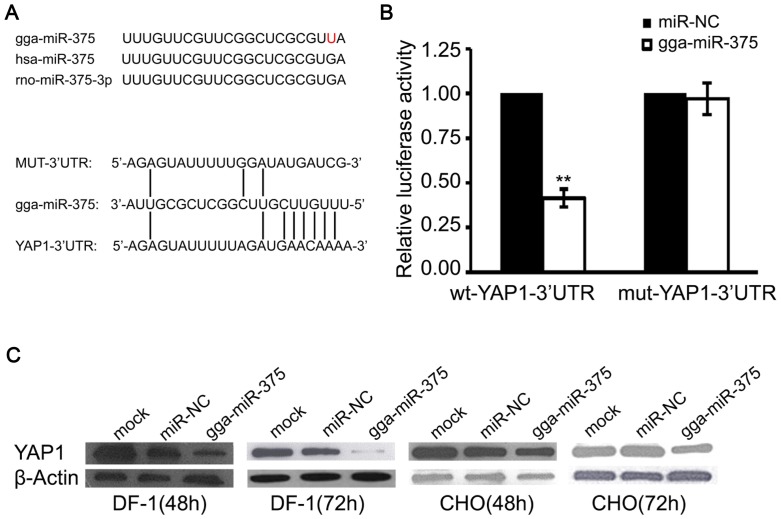
YAP1 is a direct gga-miR-375 target. (**A**) Differences in gga-miR-375, homo sapiens miR-375, and rattus norvegicus miR-375. Alignment of YAP1-3′UTR, gga-miR-375, and MUT-3′UTR, where the complementary site for the seed region of gga-miR-375 is indicated. (**B**) The regulation of luciferase activity by YAP1-3′UTR is dependent on gga-miR-375. CHO cells were co-transfected with YAP1-3′UTR-wt with either gga-miR-375 or miR-NC (left), and YAP1-3′UTR-mut with either gga-miR-375 or miR-NC (right). Columns, mean of at least three independent experiments done in duplicate; bars, SEM. **P<0.01, compared to miR-NC-transfected cells. (**C**) Ectopic expression of gga-miR-375 reduced YAP1 protein production in both DF-1 and CHO cells. β-actin levels were used as a control. Each experiment was repeated three times, and each sample was assayed in triplicate.

To explore the role that gga-miR-375 plays in ALV-J carcinogenesis, TargetScan, miRBase, and RNAhybrid algorithms were employed to search for putative cellular protein-coding gene targets of gga-miR-375. Based on TargetScan and miRBase search, YAP1 was predicted as a potential target gene of gga-miR-375 (Figure 4A). The gga-miR-375 differs from homo sapiens miR-375 and rattus norvegicus miR-375 by a single base (Figure 4A). To test whether the predicted gga-miR-375-binding sites in the 3′-UTR of YAP1 mRNA were responsible for its regulatory role, the 3′-UTR region of YAP1 was cloned downstream of a luciferase reporter gene (YAP1-3′UTR-wildtype), and co-transfected DF-1 cells with gga-miR-375 precursor, miR-NC, or NT cells. The luciferase activity of cells transfected with a gga-miR-375 precursor was significantly decreased compared to the NC (*P*<0.01; Figure 4B), indicating the mutation within the putative gga-miR-375-binding site clearly abrogated the repression of luciferase activity caused by gga-miR-375 overexpression. To further confirm YAP1 as a direct target of gga-miR-375, YAP1 protein expression was assayed 48 and 72 hours after transfection with gga-miR-375, miR-NC, or NT in DF-1 or CHO cells. The gga-miR-375 significantly suppressed the expression of YAP1 compared to miR-NC and NT (Figure 4C). These data suggested that gga-miR-375 may directly inhibit YAP1 protein production through binding to the 3′-UTR of YAP1.

### mRNA expression of *YAP1, cyclin E,* and *DIAP1* in the liver, blood, bone marrow, and spleen of ALV-J infected chickens

**Figure 5:**
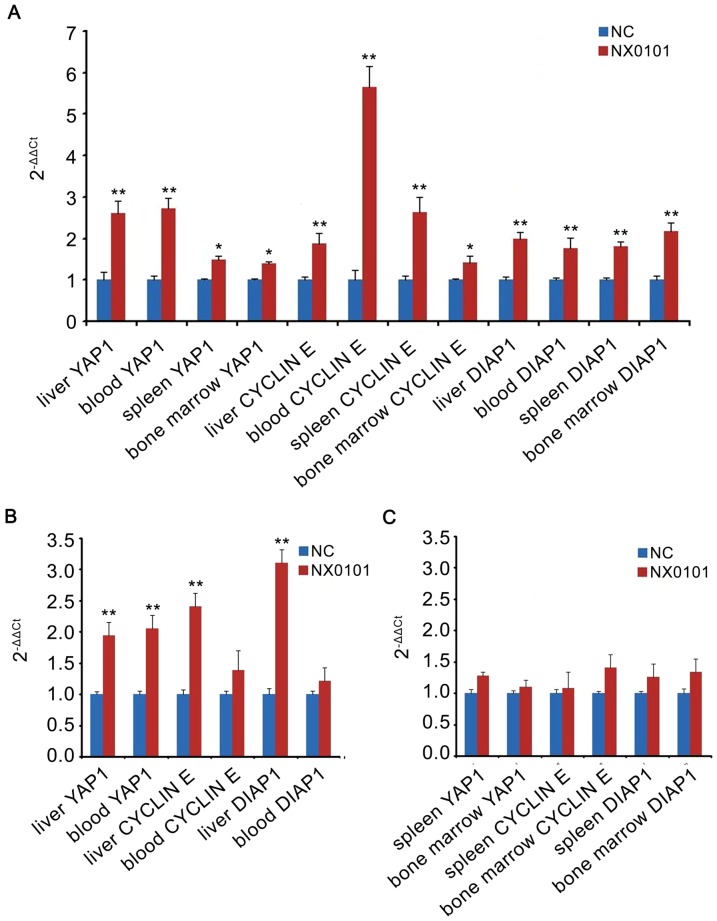
*YAP1*, *cyclin E*, and *DIAP1* gene expression in the liver, bone marrow, blood, and spleen of chickens infected with ALV-J quantified by real-time RT-PCR. (**A**) *YAP1*, *cyclin E*, and *DIAP1* gene expression at 50–60 days post-infection; *YAP1*, *cyclin E*, and *DIAP1* gene expression in the liver and blood (B) and in the spleen and bone marrow (**C**) 20–30 days post infection (**P < 0.01, *p<0.05).

Because gga-miR-375 inhibited cell proliferation and invasion and suppressed YAP1 protein production at the cellular level, we checked whether gga-miR-375 targeted Hippo signalling effector YAP1 in ALV-J infected chickens at intervals of 10 days up to 60 days. Cyclin E, a prognostic marker in other tumours, was tested in this study. DIAP1, which is associated with apoptosis, was also detection in this study. The mRNA expression of *YAP1*, *cyclin E*, and *DIAP1* during 50–60 days post infection was significantly upregulated (Figure 5A) suggesting that this period might be important for tumour formation and development. *YAP1*, *cyclin E*, and *DIAP1* expression were also significantly upregulated in livers but bone marrow and spleen at 20–30 days post-infection (Figure 5B, 5C). *YAP1* was also upregulated in blood during this period (Figure 5B). No significant differences were observed at other time points during the testing period.

## Discussion

There is substantial literature on miR-375 documenting this microRNA as a tumour suppressor in humans. However, such a role for gga-miR-375 has not been investigated to date. The data from this study showed that gga-miR-375 was significantly downregulated in liver tissue of chickens 10 weeks post ALV-J infection, which inhibited cell proliferation and promoted cell apoptosis under serum starvation. This finding suggests that YAP1 is a direct target gene of gga-miR-375. This also suggests that gga-miR-375 in chickens and miR-375 in humans are consistent on the function [40], [43], implicating mechanisms in different species and cancer types may reveal many similarities. Moreover, by directly targeting Hippo signalling effector YAP1, gga-miR-375 may directly or indirectly affect cyclin E and DIAP1 during the early stages of ALV-J infection, resulting in a range of effects on tumour development.

The role of the Hippo pathway originally defined in *Drosophila melanogaster* was to restrain cell proliferation and to promote apoptosis affecting normal cell fate and tumorigenesis [44], [45]. YAP, a transcriptional co-activator amplifier, is a pivotal effector of the Hippo pathway in mouse and human cancers; YAP1 and YAP2 are potent oncogenic drivers and independent prognostic risk factors for HCC [27], [38], [46], [47]. The importance of Hippo signalling pathway in mammalian growth control is supported by reports that transgenic overexpression of YAP, or loss of Mst1/2, leads to massive hepatomegaly and rapid progression to HCC and that YAP is amplified in some tumours and may transform immortalized mammary epithelial cells in vitro [38], [46], [48], [49], [50]. We know the size of liver and spleen in dead or sick ALV-J infected birds are enlarged to several times their normal size. However, little is known about the role of YAP1 in ALV-J induced tumours. YAP1 has several domains containing a TEAD binding region and 2 WW domains, which are DNA binding domains that function as transcriptional coactivators through interactions with DNA binding transcription factors [51], [52], [53]. YAP1 can transactivate growth-promoting genes and enhance p73-dependent apoptosis in response to DNA damage by binding to specific domains [54], [55], [56]. Here, for the first time, we show that YAP1 is a direct target of gga-miR-375. The growth of DF-1 cells was suppressed along with YAP1 expression and significantly reduced when gga-miR-375 was overexpressed, and *YAP1* appeared highly expressed in infected chickens, suggesting that YAP1 may be an oncogenic gene involved in ALV-J infection.

Organisms remove damaged or unwanted cells by an evolutionarily conserved process called programmed cell death or apoptosis [57], [58], [59]. For tumour-inducing viruses, apoptosis is a major obstacle for virus survival and the malignant transformation of host cells [60]. Overexpression of gga-miR-375 sufficiently enhanced serum starvation induced apoptosis, implying gga-miR-375 may also activate the Hippo pathway to augment apoptosis by transactivating growth-promoting genes through the TEAD binding domain of YAP1. The reason why there was different degrees of inhibition of YAP1 in DF-1 or CHO cells may be related to the mutation base (U) (Figure 4A), suggesting that for the mature RNA the miRNAs 3' end is important and provides evidence of an evolutionary relationship between the different species studies.

DIAP1 functions in the early embryo was to inhibit apoptosis [61]. In the absence of DIAP1, most cells undergo caspase-dependent apoptosis [62]. Increased DIAP1 levels are suspected to facilitate survival, as cells are very sensitive to even low levels of apoptotic inhibitors in the presence of pro-apoptotic stimuli [63], [64], [65]. As per a previous report [66], the Hippo pathway may signal through Warts to promote apoptosis by decreasing levels of the caspase inhibitor, DIAP1. Cyclin E was discovered by screening human cDNA for a rescue deficiency in G1 cyclin function in budding yeast [67]. Cyclin E is an important regulator of cell cycle progression and it reaches maximal levels of expression during the G1-to-S phase transition. This protein also exhibits specific properties that together indicate that it has an essential and rate-limiting function for allowing cells to enter into the S phase of the cell cycle [67], [68], [69], [70]. Altered expression of the cyclin E protein was reported in most breast tumour tissues and leukemia solid tumours examined to date, and aberrant levels increase with increases in tumour grade and stage [36], [71], which makes it a potential prognostic marker for some tumours. Between 50–60 days, the significant increase in levels of *DIAP1* and *cyclin E* seen in this study may serve to resist apoptosis and affect cell cycle, supporting tumour formation.

Yorkie, a Drosophila homolog of the YAP, is required for the transcription of the DIAP1 and cyclin E genes and its inactivation leads to growth arrest and apoptosis [52], [72]. As downstream genes of the Hippo pathway, *cyclin E* and *DIAP1* in mammals are significantly upregulated in the liver following the significant downregulation of gga-miR-375 in the liver, and *YAP1* is significantly upregulated. There may be a similar Hippo pathway operating in chickens. From a previous report, we know that avian leukosis infection is age-dependent; chicken resistance noticeably strengthens following growth in the first 3 weeks [73], [74], [75]. *Cyclin E* may be a prognostic marker that sharply augments, as it is similar to human *cyclin E* [71].

Together, these data show that the ALV-J virus may inhibit gga-miR-375 thus blocking the Hippo pathway and facilitate tumour progression. Additionally, the expression of related genes differed among organs; for instance, compared to the bone marrow and spleen, which are immune organs, liver and blood had higher levels of *YAP1*, *cyclin E*, and *DIAP1* expression from 20–30 days post infection. During the first 8 weeks post ALV-J infection, transformed follicles and histopathological changes are detected in 82% of the susceptible animals [76]. In this study, all tested mRNA expression was significantly upregulated during days 50–60, suggesting that this period might be a key time-point for tumour formation and development.

In poultry, ALV is the most common naturally occurring avian retroviral infection, and it causes neoplastic diseases and other production problems. As the virus infection spreads by both vertical and horizontal transmission that can have a long latency period and cause fatal damage, studies investigating the control of avian leukosis are difficult but also necessary. Our data shows that gga-miR-375 directly targets YAP1, induces cell apoptosis, and weakens the Hippo pathway, suggesting that ALV-J viruses might inhibit gga-miR-375 to influence cell proliferation, invasion, and apoptosis and subsequently affect normal cell fate and tumorigenesis. Although there is no available ALV cancer cell line, transfection with synthetic gga-miR-375 oligonucleotides as well as other approaches designed to increase endogenous gga-miR-375 together with follow-up tests of long term animal infection are needed in further studies.

## Acknowledgements

We thank Zhizhong Cui at Shandong Agricultural University for kindly providing the NX0101 strain of ALV-J for this study.
